# Supplementary material for: Experimentally revealing anomalously large dipoles in the dielectric of a quantum circuit
Source: Sci Rep. 2022 Oct 10;12:16960. doi: 10.1038/s41598-022-21256-7 (PMC9551083; doi:10.1038/s41598-022-21256-7)
Supplement: Supplementary file 1 — Supplementary Information 1. [file 41598_2022_21256_MOESM1_ESM.pdf]

Experimentally revealing anomalously large dipoles in the dielectric of a quantum circuit,
Yu *et al.*

SUPPLEMENTARY INFORMATION
Experimentally revealing anomalously large dipoles in the dielectric of a quantum circuit

Liuqi Yu,[1,2] Shlomi Matityahu,[3,4] Yaniv J. Rosen,[5] Chih-Chiao Hung,[1,2] Andrii Maksymov,[6] Alexander L. Burin,[6] Moshe Schechter,[3] and Kevin D. Osborn[1,7]

[1]*Laboratory for Physical Sciences, University of Maryland, College Park, MD 20740, USA*

[2]*Department of Physics, University of Maryland, College Park, MD 20742, USA*

[3]*Department of Physics, Ben-Gurion University of the Negev, Beer Sheva 84105, Israel*

[4]*Institut für Theorie der Kondensierten Materie,*
Karlsruhe Institute of Technology, 76131 Karlsruhe, Germany

[5]*Lawrence Livermore National Laboratory, Livermore, California 94550 USA*

[6]*Department of Chemistry, Tulane University, New Orleans, LA 70118, USA*

[7]*Joint Quantum Institute, University of Maryland, College Park, MD 20742, USA*

CONTENTS

I. Additional details of the experimental setup 2

II. Input microwave power calibration 3

III. Extraction of loss from S_{21} 4

References 5

I. ADDITIONAL DETAILS OF THE EXPERIMENTAL SETUP

The measurements are performed in a dilution refrigerator at a temperature of less than 20 mK. Supplementary Figure 1 shows the schematic of the detailed wirings of the measurement setup. A vector network analyzer (VNA) is used to carry out the S_{21} measurements. The input line is heavily filtered with multiple attenuators at different stages. An additional 12 GHz K&L low-pass filter is added. The output signal is routed through two low-noise circulators ($4-12$ GHz) which are thermally anchored at mixing chamber plate. The output signal is amplified by a high electron mobility transistor (HEMT) at 4 K. For the bias line, a -10 dB attenuator is added at room temperature. A 12 GHz K&L low-pass filter and a RC filter are placed at mixing chamber stage, which gives a cut-off frequency of 10 MHz. A time-varying bias voltage as a triangular waveform is applied. The maximum bias is $E_\mathrm{b} = 0.44\,\mathrm{V}/\mu$ m, and the fastest applied bias frequency is $f_b = 4.5$ MHz. In previous related measurements, the time-dependent loss, particularly when the bias rate changes sign, was studied[1]. It is known that the change to the measured average loss is negligible. The base temperature of the dilution refrigerator remains below 20 mK throughout all measurements, such that no significant heating from bias leakage current is observed.

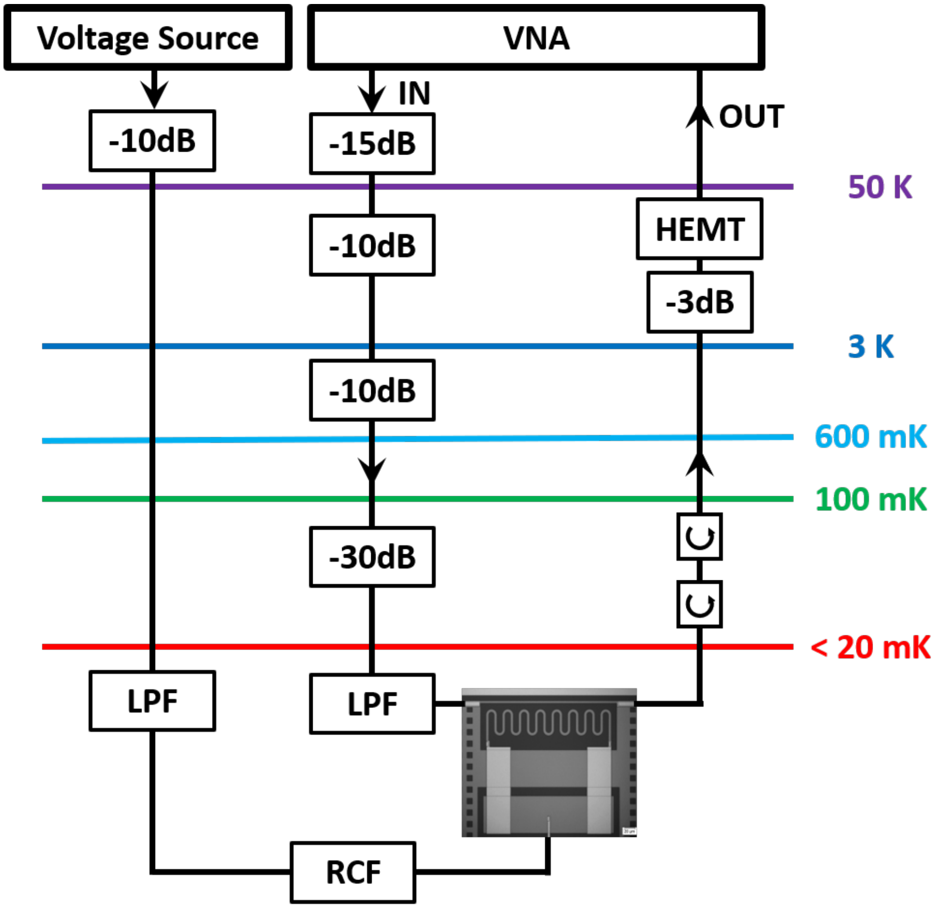

1. Schematic of the experimental wiring for measuring the resonator.

II. INPUT MICROWAVE POWER CALIBRATION

To determine the input power on chip due to attenuation in the input line, throughput S_{21}'s (bypassing the mounted device) at various source powers have been measured at room temperature. This is particularly important to accurately determine the average photon number in the resonator. The attenuation depends on the signal frequency and the source power from the VNA. Four relatively high source power (10 dBm, 0 dBm, −10 dBm and −20 dBm) are used to obtain sufficient signal to noise ratio. As shown in Supplementary Fig. 2a, linear fits are conducted on all four semilog S_{21} plots. The frequency dependence is obtained by averaging the four slopes at different applied source powers. As shown in Supplementary Fig. 2b, the source power dependence of the input power at zero frequency is determined by plotting the intercepts as a function of the source powers. The obtained slope is one, which suggests any nonlinearities in the system associated with the power is negligible. Therefore, the eventual input power at mixing chamber can be expressed as

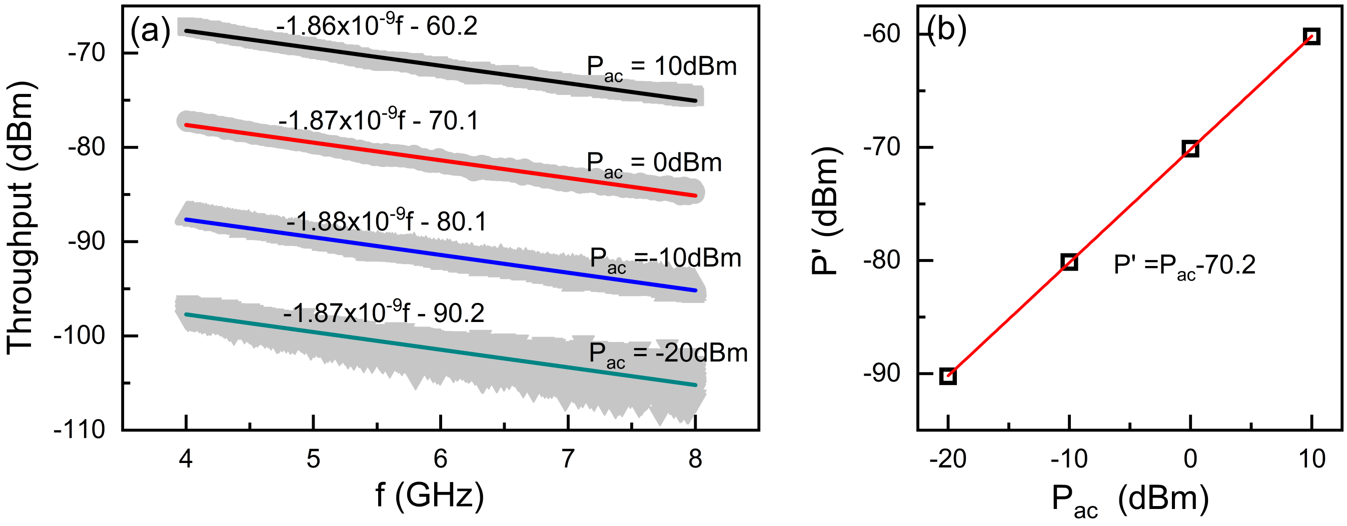

2. Input microwave power calibration. **a** Throughput calibration measurements of microwave source power at room temperature. **b** Plot and linear fit of the intercepts from the fits in **a**.

$$P = -1.87 \times 10^{-9} f + P_{\text{ac}} - 70.2 \quad (1)$$

This expression is used to calibrate the photon number on the measured resonator throughout the main manuscript.

III. EXTRACTION OF LOSS FROM S_{21}

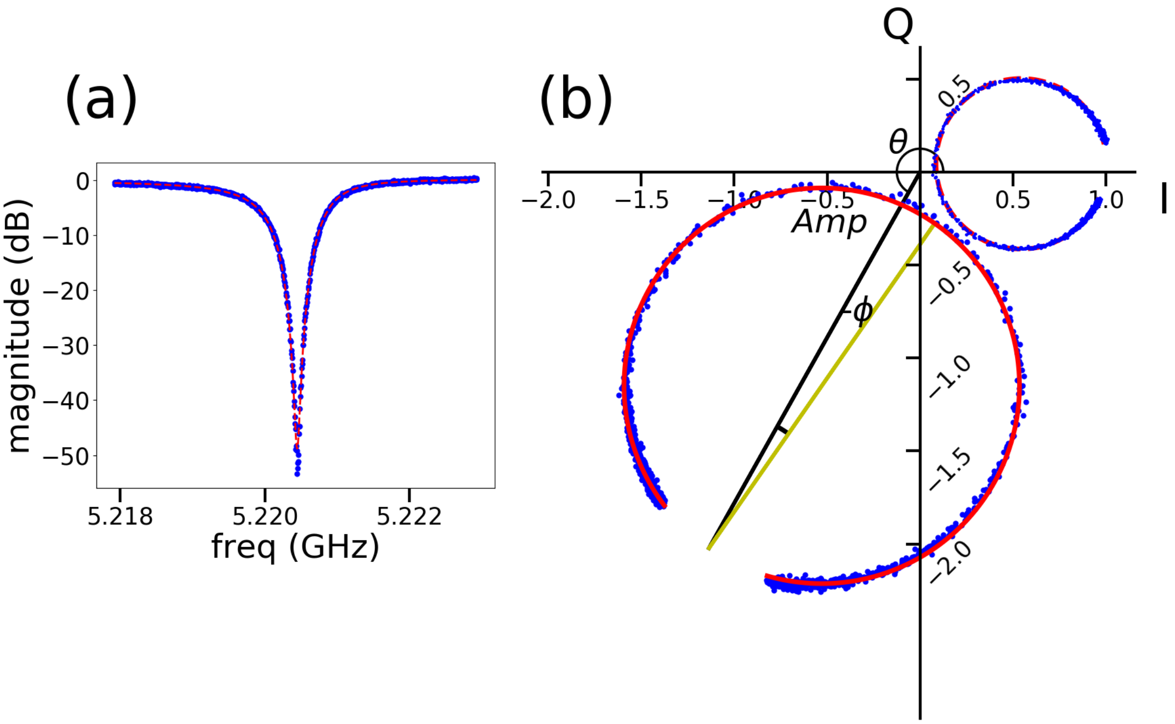

3. Fitting of loss from S_{21}. a An example of magnitude of $|S_{21}|$ vs frequency measured at $P_{\mathrm{ac}} = -29\,\mathrm{dBm}$. **b** Raw S_{21} data (blue dots) are plotted in IQ plane in the lower left. The red curve is a fitting of Supplementary Eq. 2. The black line represents the scaling of amplitude and the rotation from $(1,0)$. The yellow line represents a ϕ rotation from a small impedance mismatch. We plot the data with adjusted amplitude and electric delays on the right.

The quality factor extraction is following the fitting function[2]

$$S_{21} = Amp \times e^{i\theta} \times \frac{1 - Q|\hat{Q}_{\mathrm{e}}^{-1}|e^{i\phi}}{1 + 2iQ\frac{\omega-\omega_0}{\omega_0}}. \tag{2}$$

The Amp and θ are the adjusted parameters for the raw data due to the attenuations, amplifications, and electric delays in both input and output line, and Q is the total quality factor. Because of the impedance mismatch at two ports, $\hat{Q}_{\mathrm{e}}$ is the external quality factor and also a complex number, and ϕ is the angle of $\hat{Q}_{\mathrm{e}}^{-1}$. Fig. 3a shows an exemplary S_{21} data (magnitude vs frequency) measured at $P_{\mathrm{ac}} = -29\,\mathrm{dBm}$. The corresponding IQ plot of the data are shown in Fig. 3b: The lower left curve (blue dots) and the right curve (blue dots) are data before and after the adjusted Amp and θ. From the fitting, we extract $Q_{\mathrm{e}} = 1/Re\{\hat{Q}_{\mathrm{e}}^{-1}\} \approx 7300$, $Q_{\mathrm{i}} \approx 9000$, and $\phi = 5.7°$ when the input photon number $n = 1422$.

Supplementary References

[1] Khalil, M. S. et al. Landau-Zener population control and dipole measurement of a two-level-system bath. *Phys. Rev. B* **90**, 100201 (2014).

[2] Khalil, M. S., Stoutimore, M. J. A., Wellstood, F. C. & Osborn, K. D. An analysis method for asymmetric resonator transmission applied to superconducting devices. *J. Appl. Phys.* **111**, 054510 (2012).
